# A network analysis reveals the interaction between fear and physical features in people with neck pain

**DOI:** 10.1038/s41598-022-14696-8

**Published:** 2022-07-04

**Authors:** Valter Devecchi, Ahmed Alalawi, Bernard Liew, Deborah Falla

**Affiliations:** 1 Centre of Precision Rehabilitation for Spinal Pain (CPR Spine), School of Sport, Exercise and Rehabilitation Sciences, University of Birmingham, Birmingham, B15 2TT UK; 2 Physical Therapy Department, College of Applied Medical Sciences, Umm Al-Qura University, Makkah, 24382 Saudi Arabia; 3 School of Sport, Rehabilitation and Exercise Sciences, University of Essex, Colchester, CO4 3SQ UK

**Keywords:** Musculoskeletal system, Neurophysiology

## Abstract

Although neck pain is known to be a complex and multifactorial condition characterised by the interplay between physical and psychological domains, a comprehensive investigation examining the interactions across multiple features is still lacking. In this study, we aimed to unravel the structure of associations between physical measures of neuromuscular function and fear of movement in people with a history of neck pain. One hundred participants (mean age 33.3 ± 9.4) were assessed for this cross-sectional study, and the neuromuscular and kinematic features investigated were the range of motion, velocity of neck movement, smoothness of neck movement, neck proprioception (measured as the joint reposition error), and neck flexion and extension strength. The Tampa Scale for Kinesiophobia was used to assess fear of movement. A network analysis was conducted to estimate the associations across features, as well as the role of each feature in the network. The estimated network revealed that fear of movement and neuromuscular/kinematic features were conditionally dependent. Higher fear of movement was associated with a lower range of motion, velocity, smoothness of neck movement, neck muscle strength, and proprioception (partial correlations between − 0.05 and − 0.12). Strong interactions were also found between kinematics features, with partial correlations of 0.39 and 0.58 between the range of motion and velocity, and between velocity and smoothness, respectively. The velocity of neck movement was the most important feature in the network since it showed the highest strength value. Using a novel approach to analysis, this study revealed that fear of movement can be associated with a spectrum of neuromuscular/kinematic adaptations in people with a history of neck pain.

## Introduction

The concept of complexity is being increasingly adopted to describe the multidimensional nature of spinal pain within the biopsychosocial framework^1–3^. This view has been emphasised because complexity emerges from the dynamic interactions across its multiple components^4^. Given the complexity of spinal pain including neck pain, new approaches are available to circumvent some of the limitations associated with a reductionist biomedical view (e.g., focus on a single domain overlooking cross-domain interactions), resulting in a better understanding of the nature of chronic pain^1,5^.

The relationship between kinesiophobia i.e. fear of movement, and neuromuscular control has frequently been investigated in people experiencing pain to understand how physical and psychological components are interrelated, and different theories have been proposed^6,7^. The fear-avoidance model suggests that fear of movement and catastrophic thoughts result in movement avoidance, deconditioning, and ultimately disability^7^. It has been shown that fear of movement mediates the relationships among catastrophizing, pain intensity, and disability in people with neck pain^8,9^, yet any interactions with movement avoidance and deconditioning are less clear^10,11^. Besides changes in physical activity levels, people with neck pain often present with several specific changes in physical function including reduced range and speed of movement^12,13^, altered variability and smoothness of movement^14,15^, impaired proprioception^16,17^, and reduced neck muscle strength^18,19^, which potentially could all be influenced by fear of movement.

Although previous studies have investigated the relationship between neuromuscular/kinematic features and fear of movement in people with neck pain, this work was largely limited to investigations of neck movement where simple correlations were carried out between a single measure of physical function and fear of movement^20^; an approach which oversimplifies the complexity and variability of physical features in people with neck pain. Considering the importance of restoring physical fuction to improve pain and disability in people with neck pain^21–23^, the investigation of the interactions between physical features is necessary to understand if any transfer effect may be obtained from the treatment of a feature to one another.

Network analysis (NA), a methodology applied to study complex systems^24^, is a more appropriate approach to examine the interaction between fear of movement and neuromuscular/kinematic features in people with pain since it can be used to quantitatively assess and graphically describe multiple interactions. In this regard, the presence of strong and multiple interactions across features can help to identify potential targets for rehabilitation. Furthermore, one aim of a NA is to discern causal relationships among features by revealing if they are conditionally dependent, and, although correlation is not causation, NA can represent the first step in this direction^25^. The use of NA in pain-related research has increased in the last few years, albeit with a focus limited to the interactions between psychological features^26,27^. To the best of our knowledge, this is the first study to conduct a comprehensive investigation of the relationship between kinesiophobia and neuromuscular/kinematic features in people with neck pain by applying a NA. The present study aimed to provide a greater understanding of neck pain complexity taking into account physical and psychological factors.

## Methods

### Study design

This cross-sectional study was performed at the University of Birmingham, United Kingdom, and was conducted following the Declaration of Helsinki. Ethical approval was obtained from the University of Birmingham Ethics Committee (ERN_19-0564). All participants provided written informed consent. The report of this study follows the “Strengthening The Reporting of Observation studies in Epidemiology” (STROBE checklist)^28^ and the reporting standards for network analyses^29^.

### Participants

Men and women aged between 18 and 65 years, with and without a history of neck pain were recruited for this study. People with a history of spinal or shoulder surgery, neck or shoulder injury that resulted in a fracture, current neuropathies/radiculopathies, neurological deficits, rheumatic condition, or pregnancy were excluded. Additionally, people who had participated in a neck or shoulder exercise programme in the past three months were excluded.

Participants with a history of neck pain were eligible if they experienced chronic or recurrent episodes of neck pain. Participants with recurrent neck pain episodes were included following the criteria reported by Stanton et al.^30^, specifically, people (i) who experienced two or more episodes of neck pain during the previous 12 months and (ii) reported a pain score during an episode of at least 2 out of 10 on the numerical rating scale (NRS, 0–10). Participants with chronic neck pain were included if they (i) experienced pain lasting three months or more; (ii) reported a pain intensity over the previous 24 h equal or greater than 2 out of 10 on the NRS, and (iii) a minimum score of 10 out of 50 on the Neck Disability Index (NDI)^31^. Participants without a history of neck pain were eligible if had no current neck pain and no history of neck or shoulder pain that required treatment from a healthcare professional.

A convenience sample was considered for this study given the exploratory nature of this investigation and because criteria necessary to define the adequate sample size in a NA are still an object of discussion^32^. The recruitment of a heterogeneous population of people with and without a history of neck pain allowed us to obtain greater variance in the values related to fear of movement and neuromuscular/kinematic features.

### Procedure

All participants completed the Tampa Scale for Kinesiophobia (TSK), a 17-item questionnaire used to assess pain related-fear and information about fear of movement and avoidance behaviours^33^. Participants with a history of neck pain were also required to complete the NRS and the NDI to record their average neck pain over the previous 24 h and evaluate their disability, respectively^31,34^.

Neck movements were quantified using wearable Inertial Measurement Units (Research PRO IMU, Noraxon, USA) with a sampling rate of 100 Hz. Sensors were placed via double-sided tape on the forehead, upper, and lower thoracic spine (T1 and T12, respectively). To assess neck kinematics and proprioception, participants were asked to adopt a natural upright position while sitting, with their arms supported, their feet on the ground, and without touching the backrest. Then, participants were instructed to perform ten continuous cycles of neck flexion/extension moving as far as possible and with a self-paced natural speed. Then, the same procedure was applied to assess neck rotation and lateral flexion movements. Overall, thirty cycles were performed, ten for each plane of motion (i.e., flexion/extension, rotation, lateral flexion) with one-minute rest provided between movement directions.

Once the assessment of neck kinematics was completed, proprioception was evaluated using the cervicocephalic relocation test^35^. From a self-selected neutral head posture while sitting (starting position), participants were asked to perform full right or left neck rotation with their eyes closed and then return, as accurately as possible, to the starting position. Participants were allowed to move at a preferred speed that was comfortable for them. After each trial, the participants were asked to return to a self-selected neutral head posture with their eyes open. To identify the ending position along the movement waveform (i.e., neck rotation), a digital marker was added by the physiotherapist when the participant verbally reported being in the neutral position (after performing rotation) again. The starting position, instead, was identified as the one that immediately preceded the rotation movement. In total, six repetitions were performed, alternatively, to the right and left side (same order among all participants). A resting period of two minutes was provided between repetitions. Signals of trunk kinematics were monitored online by one physiotherapist to ensure that no trunk rotation movements were present during the task.

Finally, participants performed two maximal voluntary contractions (MVC) of isometric neck extension and flexion. This task was conducted in the Multi-Cervical Unit (BTE Technologies)^36^. After a few low force contractions to familiarize the participant with the task, two MVCs were performed in both extension and flexion. The MVCs were of four seconds duration, separated by 2 min of rest. The maximal peak force was recorded for each direction.

### Data processing and feature extraction

From the investigated tasks, the objective outcomes of interest were range of motion (ROM), velocity of movement, smoothness, proprioception, and isometric neck muscle strength.

#### Neck kinematics

Raw data of anatomical angles were retrieved from the inertial measurement system and exported in MATLAB (R2020a, Mathworks) for offline analyses. All kinematics data were low-pass filtered with a 10th order Butterworth filter and with a 10 Hz cut-off frequency^15^.

The procedure for the kinematic analysis of neck movements is explained here for the flexion/extension movement, but the same approach was applied for rotation and lateral flexion movements (different waveforms). From the angle waveform, each peak value represents the maximal ROM during the corresponding cycle. From the movement peaks, segments of the angle waveform were collected to obtain full-range flexion and extension movements. Velocity was computed by numerical differentiation of the angle waveform (neck flexion/extension movement). During each cycle, mean velocity and smoothness were calculated between the start and end of the movement, defined as the time when the velocity passed the threshold of 5% computed from the peak velocity value^15^. Mean velocity for every direction was obtained averaging the mean angular velocity extracted from each cycle of movement.

The assessment of smoothness of movement was conducted using the spectral arc length (SPARC), an outcome measure computed from the power spectrum of the velocity signal^37^. Lower SPARC values indicate less movement smoothness and greater frequency composition. SPARC values were extracted for each movement direction and results were used for subsequent analysis.

#### Proprioception

The joint reposition error in the primary plane of movement (rotation) was computed averaging the absolute errors of six trials (three for each direction). The start and the end position of the movement were visually detected and used to calculate the absolute error of one trial^38^.

#### Isometric neck strength

Peak values (one per direction) obtained from the two trials of MVC in flexion and extension were used for subsequent analysis.

### Statistical analysis

All statistical analyses were conducted using R software (version 4.0.2)^39^. Demographic characteristics and outcome measures are descriptively reported using mean and standard deviation. Questionnaire scores were evaluated and reported according to their respective guidelines^31,33^. Pearson correlations were performed between the features included in the NA.

#### Network analysis

Before conducting the NA, kinematics features were averaged across all movement directions (flexion, extension, right and left rotation, right and left lateral flexion) and collapsed in three variables representing ROM, speed, and smoothness of movement for each participant (Table 1). The same approach was conducted for strength values (flexion and extension) which were averaged as a single variable. Overall, potential features which were included in the network were *ROM*, *velocity*, *SPARC*, *JPE*, *strength*, *TSK* (Table 1). Nonparanormal transformation was conducted before estimating the structure of the network using *npn* function (*huge* package)^40,41^. One hundred complete cases were included in the NA.

**Table 1:** Features and nodes included in the network analysis.

Features		Nodes
**Maximal range of motion**		ROM^a^
Flexion	Extension	
R rotation	L rotation	
R lateral flexion	L lateral flexion	
**Mean velocity**		Velocity^a^
Flexion	Extension	
R rotation	L rotation	
R lateral flexion	L lateral flexion	
**Smoothness of movement**		SPARC^a^
Flexion	Extension	
R rotation	L rotation	
R lateral flexion	L lateral flexion	
Proprioception		JPE
**Strength**		Strength^a^
Flexion		
Extension		
Fear of movement		TSK

*JPE* Joint position error, *ROM* Range of motion, *SPARC* Spectral arc length, *TSK* Tampa scale for kinesiophobia.

^a^Average values obtained from the directions of movement reported in the first column.

NA was performed with the R-package *bootnet*^42^. In a network, features are graphically represented as nodes, and the partial correlation coefficients that define the interactions among them are represented by edges^42,43^. Partial correlations are also defined as conditional (in)dependence associations because they are computed after conditioning on all other nodes^43^. Hence, the presence or absence of an edge between two nodes indicates a conditional dependency or independency, respectively^43^. Edges in a network are graphically reported with different colours to represent positive versus negative correlations, and their thickness depends on the strength of the correlation (edge weight).

Although partial correlations protect against spurious associations (i.e. false positive), most of the relationships identified are usually nonzero and the network still results in a dense structure. Therefore, a common regularization technique in NA, “graphical least absolute shrinkage and selection operator” (glasso), was applied to set weak coefficients to zero and further controlling for spurious correlations^42,43^. The glasso algorithm allows to select the complexity of the network and optimize its structure by minimizing the Extended Bayesian Information Criterion (EBIC).

After estimating the structure of the network, centrality indices including strength, closeness, and betweenness were evaluated to investigate how well each feature is associated with the others^42^. Briefly, the strength of a node defines how strongly it is directly connected to the other nodes; a feature with a high strength commonly has a large impact on the others. Betweenness quantifies the importance of a node in the network considering how often it is crossed by the shortest paths that connect the other two nodes. Closeness quantifies the distance between a node to all the others, or, in other words, how strongly a node is indirectly connected to the others. Therefore, centrality indices computed for each node can provide valuable information to understand the role of included features in the population of interest. From a clinical perspective, it means that the treatment of a feature high in strength could deserve particular attention because its improvement is likely to impact other interrelated features. Also, if such a feature shows high values in betweenness and closeness it means that its impact is on most of the other features included in the estimated network.

Stability of centrality indices and edge-weight accuracy were assessed using non-parametric bootstrapping (resample with replacement, n = 1000). Stability is ensured when the indices retain a correlation of at least 0.7 after dropping a proportion of cases equal to or greater than 0.5 (correlation stability indices, CS_cor=0.7_)^42^. Edge-weight accuracy is obtained from edge-weight bootstrapped confidence intervals (CIs).

Further analyses (Supplementary File 1) were conducted to facilitate the replicability of results in future studies and provide an estimate of the required sample size. To this end, the *replicatSimulator* and *netSimulator* functions of the R-package *bootnet* were used.

## Results

Of one hundred participants included in the analysis, 15 had no history of neck pain and 85 reported recurrent (n = 65) or chronic (n = 20) neck pain. Their demographic characteristics are reported in Table 2. Idiopathic and trauma-induced neck pain were the causes of neck pain in 57 and 28 participants, respectively.

**Table 2:** Demographic characteristics (mean ± SD).

	No history of neck pain (*n* = 15)	History of neck pain		
		Recurrent neck pain (*n* = 65)	Chronic neck pain (*n* = 20)	Total (*n* = 85)
Age	31.4 ± 5.5	33.9 ± 10.3	33.0 ± 8.9	33.6 ± 9.9
Gender (F/M)	8/7	41/24	14/6	55/30
Weight (kg)	69.2 ± 14.7	74.9 ± 18.5	65.5 ± 13.7	72.7 ± 17.8
Height (cm)	173.5 ± 8.6	170.9 ± 9.1	167.0 ± 9.7	169.9 ± 9.3
Pain intensity (NRS, 0–100)	–	–	53.4 ± 18.2	12.6 ± 24.4
NDI (0–50)	–	4.5 ± 3.9	16.5 ± 6.1	7.3 ± 6.8
TSK (17–68)	29.3 ± 4.4	34.9 ± 5.9	35.2 ± 8.0	35.0 ± 6.4

*NDI* Neck disability index, *TSK* Tampa scale for kinesiophobia.

Participants with recurrent neck pain were asymptomatic and presented no neck disability (NDI: 4.5 ± 3.9). Instead, participants with chronic neck pain had moderate pain (NRS: 53.4 ± 18.2) and disability (NDI: 16.5 ± 6.1). Fear of movement was similar between participants with recurrent and chronic neck pain (TSK: 34.9 ± 5.9 and 35.2 ± 8.0, respectively). Participants without a history of neck pain had no to mild fear of movement (TSK: 29.3 ± 4.4). Descriptive statistics of neuromuscular/kinematic features in people with and without a history of neck pain are summarised in Table 3. Pearson correlations between features included as nodes in the NA are presented in Table 4.

**Table 3:** Mean and SD of neuromuscular/kinematic features included in the NA.

	No history of neck pain (*n* = 15)	History of neck pain (*n* = 85)
JPE (°)	7.00 ± 4.50	6.10 ± 3.70
ROM (°)	53.0 ± 6.8	49.7 ± 6.7
Velocity (°/s)	76.7 ± 21.5	61.6 ± 20.3
SPARC	− 1.61 ± 0.08	− 1.68 ± 0.11
Strength (N)	108.5 ± 56.38	81.8 ± 30.7

*JPE* Joint position error, *ROM* Range of motion, *SPARC* Spectral arc length.

**Table 4:** Matrix of Pearson correlations between the investigated features.

	TSK	JPE	ROM	Velocity	SPARC	Strength
TSK	–					
JPE	0.16	–				
ROM	− 0.24*	− 0.01	–			
Velocity	− 0.27**	0.18	0.46**	–		
SPARC	− 0.21*	0.16	0.14	0.65**	–	
Strength	− 0.19*	0.24*	0.07	0.20*	0.08	–

*JPE* Joint position error, *ROM* Range of motion, *SPARC* Spectral arc length, *TSK* Tampa scale for kinesiophobia.

*p ≤ .05; **p < .01.

**Figure 1:**
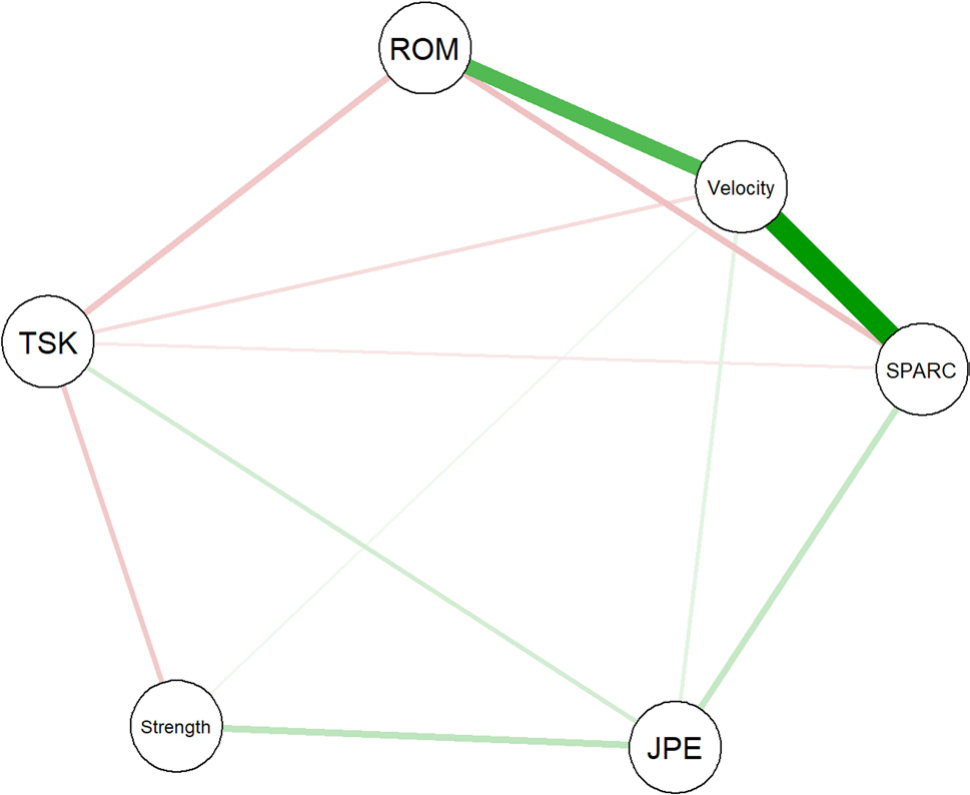
Network estimated from neuromuscular/kinematic features (*JPE* Joint position error, *ROM* Range of motion, *SPARC* Spectral arc length; *Strength; Velocity*, velocity of neck movements) and fear of motion (*TSK*). Positive and negative partial correlation coefficients are displayed in green and red, respectively. The thickness of the edges represents how strong the relationships between nodes are.

**Figure 2:**
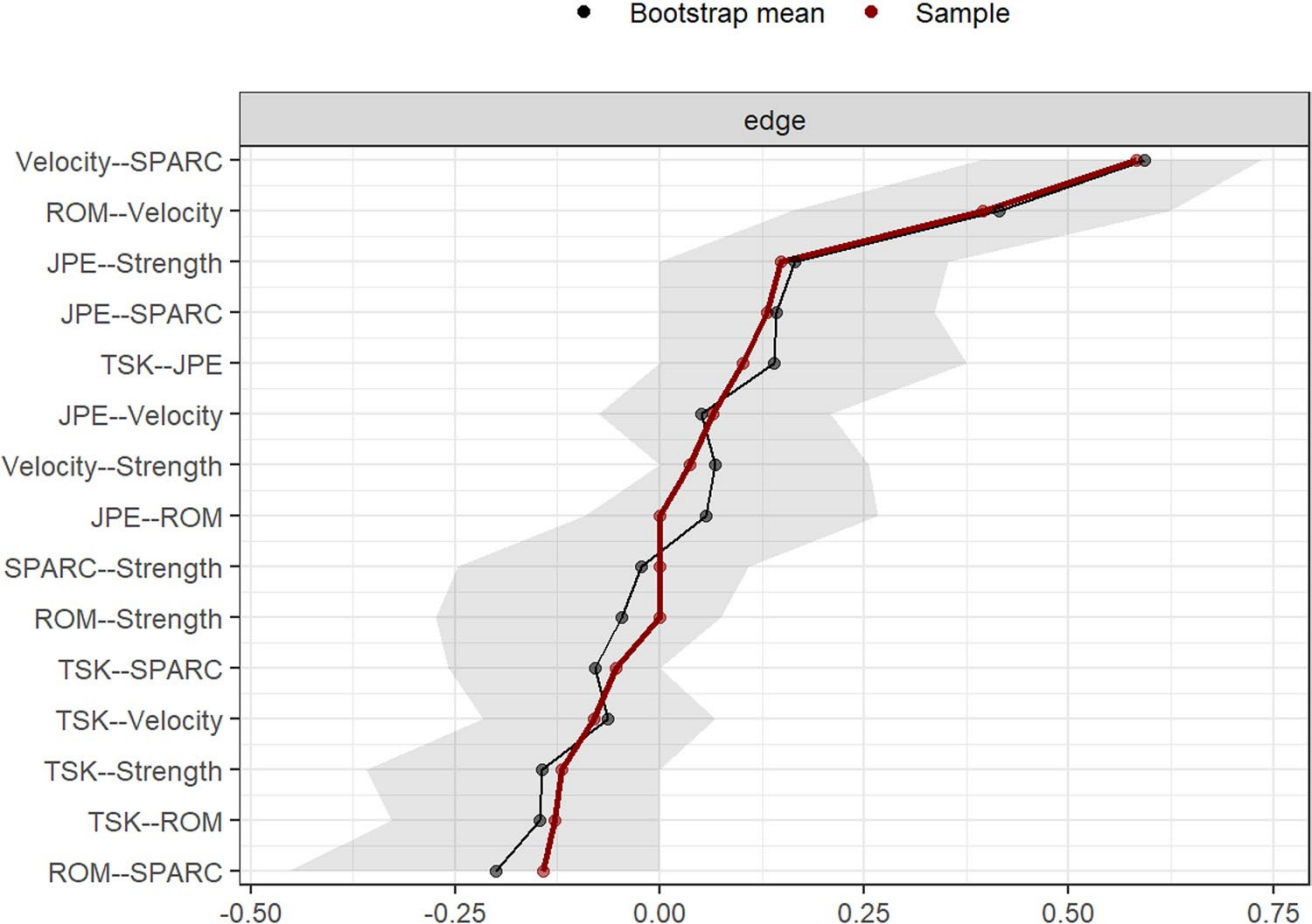
Edge weights (based on original and bootstrapped samples) along with their variability (bootstrapped confidence intervals, grey area) are reported. *SPARC* Spectral arc length, *TSK* Tampa scale for kinesiophobia, *ROM* Range of motion.

The partial correlation network estimated from the features reported in Table 1 is illustrated in Fig. 1. Of 15 possible edges, 12 were retained after regularization. Fear of motion (*TSK*) and *velocity* were connected to all other nodes, and the latter shares the strongest relationships in the network with *ROM* and *SPARC*. Specifically, partial correlation was 0.39 between *ROM* and *velocity*, and 0.58 between *velocity* and *SPARC*. Although the edges *ROM–velocity* and *velocity–SPARC* were defined by positive partial correlation coefficients, the edge *ROM–SPARC* was negative (− 0.14) and unexpected. Such a relationship could result from a common effect structure (see “Discussion” section). Fear of motion showed negative associations with all nodes (partial correlations between − 0.05 and − 0.12), except for a positive one with *JPE* (0.10). Edge weights and their variability are presented in Fig. 2. The high edge weights between *Velocity* and other kinematics features were also characterised by low variability. Instead, edge weights with high variability ranging between positive and negative values were set to zero (e.g., between *SPARC* and *strength*).

**Figure 3:**
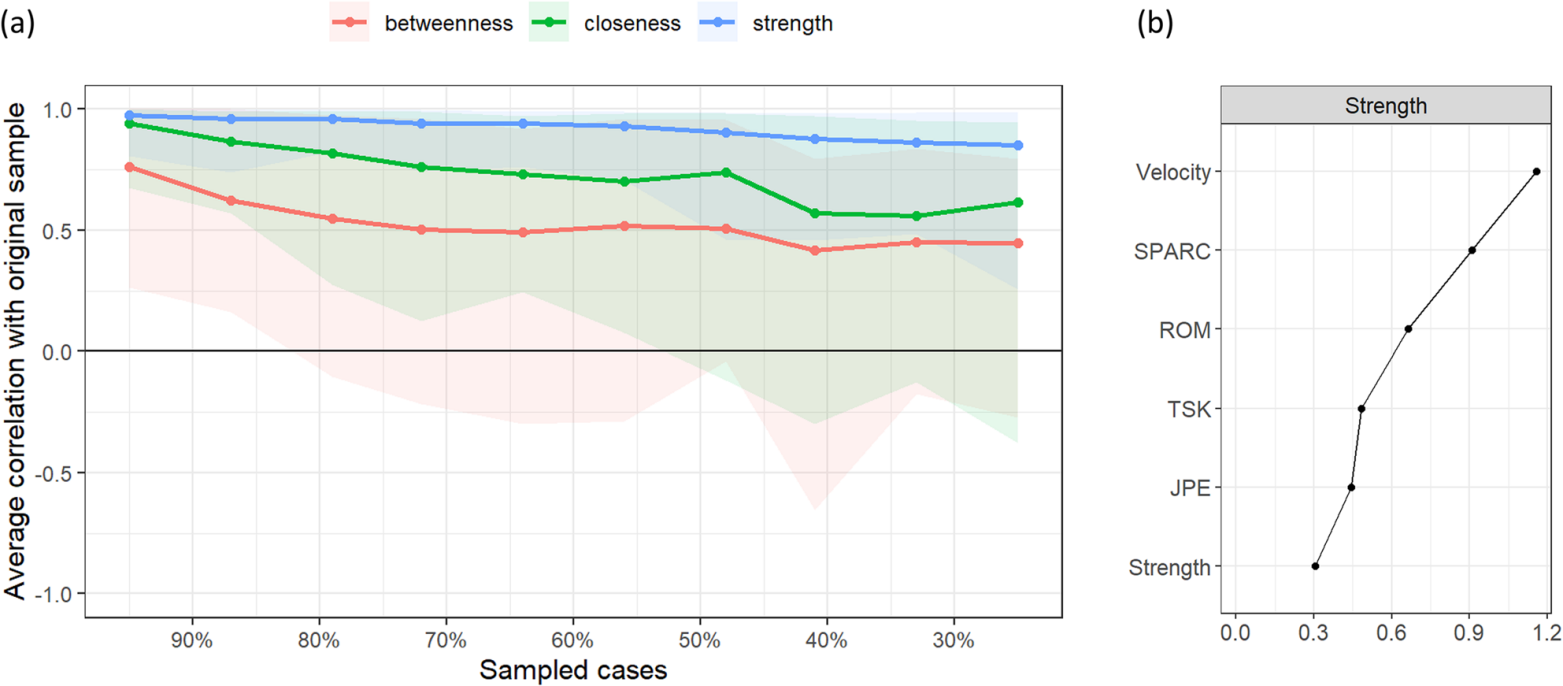
Correlation stability plot of centrality indices obtained from case-dropping bootstrap (**a**) and node centrality measure of Strength (raw coefficients). (**b**) Betweenness and closeness were not stable since their large confidence intervals and the poor correlation with original values when 50% of cases were dropped. *JPE* Joint position error, *ROM* Range of motion, *TSK* Tampa scale for kinesiophobia (fear of movement).

Based on the correlation stability indices obtained from non-parametric bootstrapping (Fig. 3), only Strength was stable (CS_cor=0.7_ = 0.52), whereas Closeness and Betweenness were extremely unstable since affected by a large CI (CS_cor=0.7_ = 0 and CS_cor=0.7_ = 0.05, respectively). *Velocity* of neck movements was the node with the highest strength value in the network because of its relationships with all other nodes and the strong associations with *ROM* and *SPARC*. Strength centrality measure is presented in Fig. 3 for all nodes. Results from simulation analyses concerning sample size estimation and replicability of findings are summarised in the Supplementary File (online).

## Discussion

The multidimensional nature of presentation inherent to people with neck pain requires an approach to assessment that can handle multiple factors simultaneously. In the present study, we introduced the use of a NA to estimate the relationships between fear of movement and neuromuscular/kinematic features in people with a history of neck pain. Findings from the estimated network revealed that higher fear of movement was associated with lower ROM, velocity, and smoothness of neck movements, as well as poorer neck proprioception and less neck muscle strength. The structure of the identified network extends the fear avoidance model by describing the interactions between kinesiophobia and objective measure of neuromuscular/kinematic features, in this case, specifically applied to people with a history of neck pain. Overall, the inclusion of different features allowed us to obtain a comprehensive overview and understand how individual physical factors were associated with fear of movement.

The relationship between fear of movement and altered kinematics in people with neck pain is extensively supported in the literature. Sarig Bahat et al. found that reduced ROM, velocity, and smoothness of neck movements were associated with higher fear of movement^20^. Similar results were also reported by Meisingset et al.^12^. In these studies, however, the high correlation among kinematic features was not considered when their associations with fear of movement were evaluated. Thus, identified relationships between one kinematic feature and fear of movement could result from a shared association with another kinematic feature. Instead, in the estimated network of partial correlation coefficients that we have presented, the associations described also remained after conditioning on all other kinematic variables, revealing that higher fear of movement was specifically associated with reduced range, velocity, and smoothness of neck movement. In other words, fear of movement and the investigated neck kinematic features were conditionally dependent.

**Figure 4:**
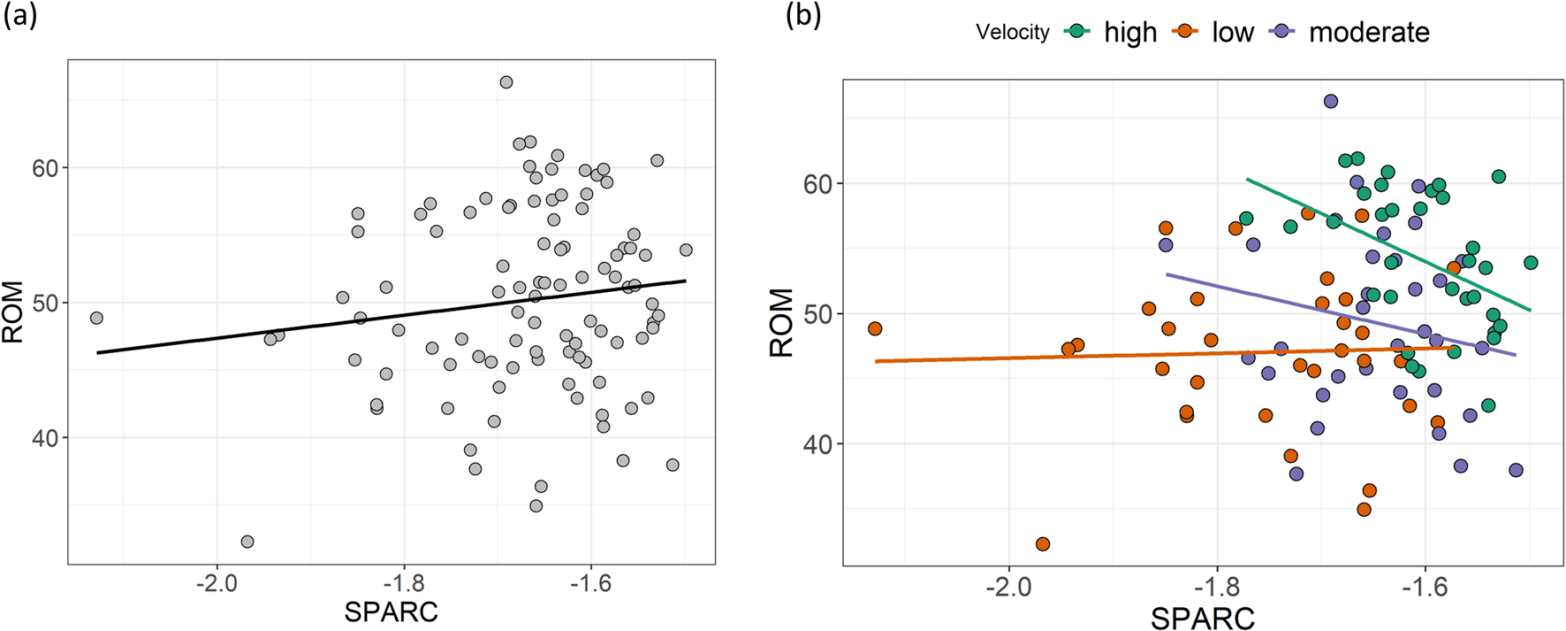
Association between ROM and SPARC before (**a**) and after (**b**) conditioning on velocity. *ROM* Range of motion (degrees), *SPARC* Spectral arc length (smoothness of neck movements).

Although the positive associations *ROM–velocity*, and *velocity–SPARC* were as expected, the negative association *ROM–SPARC* was counterintuitive if compared with findings encountered in the investigation of people with neck pain (i.e., reduced ROM, velocity, and smoothness of neck movements)^12,15^. This issue affecting the triplet of features (*ROM, velocity*, and *SPARC*) could arise because the association *ROM–SPARC* is conditioned on *velocity*. In other words, if *velocity* is considered as a confounder and data are segregated by it, the association *ROM–SPARC* is reversed (especially in people with high and moderate velocity of neck movement). A graphical illustration of this statistical phenomenon, known as Simpson’s paradox^44^, is presented in Fig. 4 where participants were divided into low, moderate, and high neck movement velocity subgroups. Furthermore, the different sign of partial and normal correlation between SPARC and ROM (− 0.13 and 0.14, respectively) further suggests the presence of a common effect and the impact of velocity on this interaction^29^.

Another relevant interaction revealed by the estimated network is the one between fear of movement and neck muscle strength. Similar to the findings by Lindstroem et al., we found a negative association between fear of movement and neck muscle strength^19^. Notwithstanding the impossibility to prove causality in the estimated undirected network, the identified relationship likely supports the fear-avoidance model which considers fear of motion as a potential driver for physical deconditioning^7^.

In addition to kinematic features and strength, higher fear of movement was also associated with poorer proprioception (larger joint position error). However, findings for this relationship, as well as on the clinical utility of sensorimotor tests in people with neck pain, are not consistent across the literature^17,45^. In this regard, the structure of a network may reveal the existence of interactions across features able to impact their assessment. Accordingly, a network might allow to identify relevant covariates that need to be considered when a measure of physical function is investigated in different groups (e.g., comparison between people with and without spinal pain). In our estimated network, for example, a large joint position error was associated not only with higher fear of movement but also with a higher speed of neck movement. One reason might be that different motor strategies are adopted by participants based on their level of fear of movement. For instance, participants with low fear of movement could perform the task with a higher velocity of movement which could impact performance, and, on the other hand, higher fear of movement could result in slower and more cautious movements. A similar hypothesis has been suggested by Goncalves and Silva^46^. To explain their finding of a negative correlation between repositioning error and catastrophizing thoughts (i.e., smaller error in people with higher catastrophising), they argued that negative beliefs about pain and movement could lead individuals to increase their cognitive attention during the task^46^.

Along with the identification of feature interactions, NA allows to evaluate specific properties of a node, commonly described by centrality indices. Of the centrality indices assessed in the estimated network (strength, betweenness, and closeness), only node strength showed adequate stability (correlation stability indices greater than 0.5). Instead, betweenness and closeness were affected by a large confidence interval which limits their interpretation, and consequently our understanding of the role of a feature in the network. This issue was also reported in other studies investigating psychological domains, leading some authors to question the utility of betweenness and closeness^47^. Of the included features, the *velocity* of neck movement showed the highest strength. In other words, a change in velocity was associated to a greater extent with a change in other features. From a clinical perspective, the velocity of neck movement might be an important feature given its associations with strength, range of motion, smoothness, and fear of movement. In support of the clinical value of the velocity of neck movement, intervention studies in people with neck pain have identified a reduction in pain, disability, and fear of movement along with improvement of neck kinematic features (mainly velocity and smoothness of movement)^21,48,49^. Interestingly, these findings were reported both after exercise and manual therapy interventions^21,48,49^.

The identified associations are following previous studies that investigated the association between kinesiophobia and multiple neuromuscular/kinematic features in people with neck pain^20,50^. Here, our results also revealed that kinesiophobia and neuromuscular/kinematic features are conditionally dependent, and future research is needed to assess the implications of such associations when influenced by interventions. Furthermore, our findings may broadly apply to adults with neck pain since we included people with recurrent and chronic neck pain, and of both idiopathic and traumatic origin. On the other hand, however, generalisability to an older population cannot be done and should be addressed in future research. Notwithstanding the relevant exploration of interactions between psychological and neuromuscular/kinematic features, our findings need to be considered with caution because of some study limitations. In the estimation of a network, there is a trade-off between exploratory and conservative approaches which usually results in dense or sparse structures, respectively. In this study, we erred on the side of exploration given the novelty of the investigation. Although this approach might estimate a network with spurious correlations, the use of *glasso* regularization allowed us to address this issue. On the other hand, a conservative approach would have reduced the sensitivity of the analysis by increasing the risk of removing true relationships. The replicability of the estimated network needs to be addressed in future studies. Replicability of networks is a nontrivial problem since it can depend on unstable findings (failure to replicate) or relevant differences between studies (failure to generalise). Here, we provided initial evidence about potential issues concerning the stability of centrality indices, as well as analyses to facilitate the estimation of sample size in future studies.

A further limitation that partially affects the interpretability of the network is represented by the reduction of features (evaluated from different directions of movement) into a single node. Nevertheless, feature reduction was necessary since the number of nodes has an impact on the estimation of a network; penalties for model regularisation and selection are computed taking into account the number of included nodes. Also, for each extra feature added into the network, the number of potential edges that need to be computed grows dramatically.

Relationships across features were described through an undirected network, hence, no inferences can be made in terms of causality. As demonstrated, however, identified associations can promote the generation of hypotheses and indicate the presence of potential causal relationships to investigate further. In this regard, data from longitudinal studies are warranted to test such causal associations and provide new insights into the dynamic relationships between physical and psychosocial features.

## Conclusion

Findings from the estimated network extend the fear-avoidance model showing the associations between kinesiophobia and neuromuscular/kinematic features in people with a history of neck pain. Specifically, higher values of kinesiophobia were associated with lower range of motion, velocity, smoothness of neck movements, proprioception, and neck strength. NA is a promising tool to unravel the complexity and interaction between physical and psychological features in people with neck pain. Future longitudinal studies should consider the use of NA to understand how these cross-domain interactions change over time.

## Supplementary Information

Supplementary Information.
