# Supplementary material for: A network analysis reveals the interaction between fear and physical features in people with neck pain
Source: Sci Rep. 2022 Jul 4;12:11304. doi: 10.1038/s41598-022-14696-8 (PMC9253153; doi:10.1038/s41598-022-14696-8)
Supplement: Supplementary file 1 — Supplementary Information. [file 41598_2022_14696_MOESM1_ESM.docx]

**Supplementary Information -** Network Estimation Performance


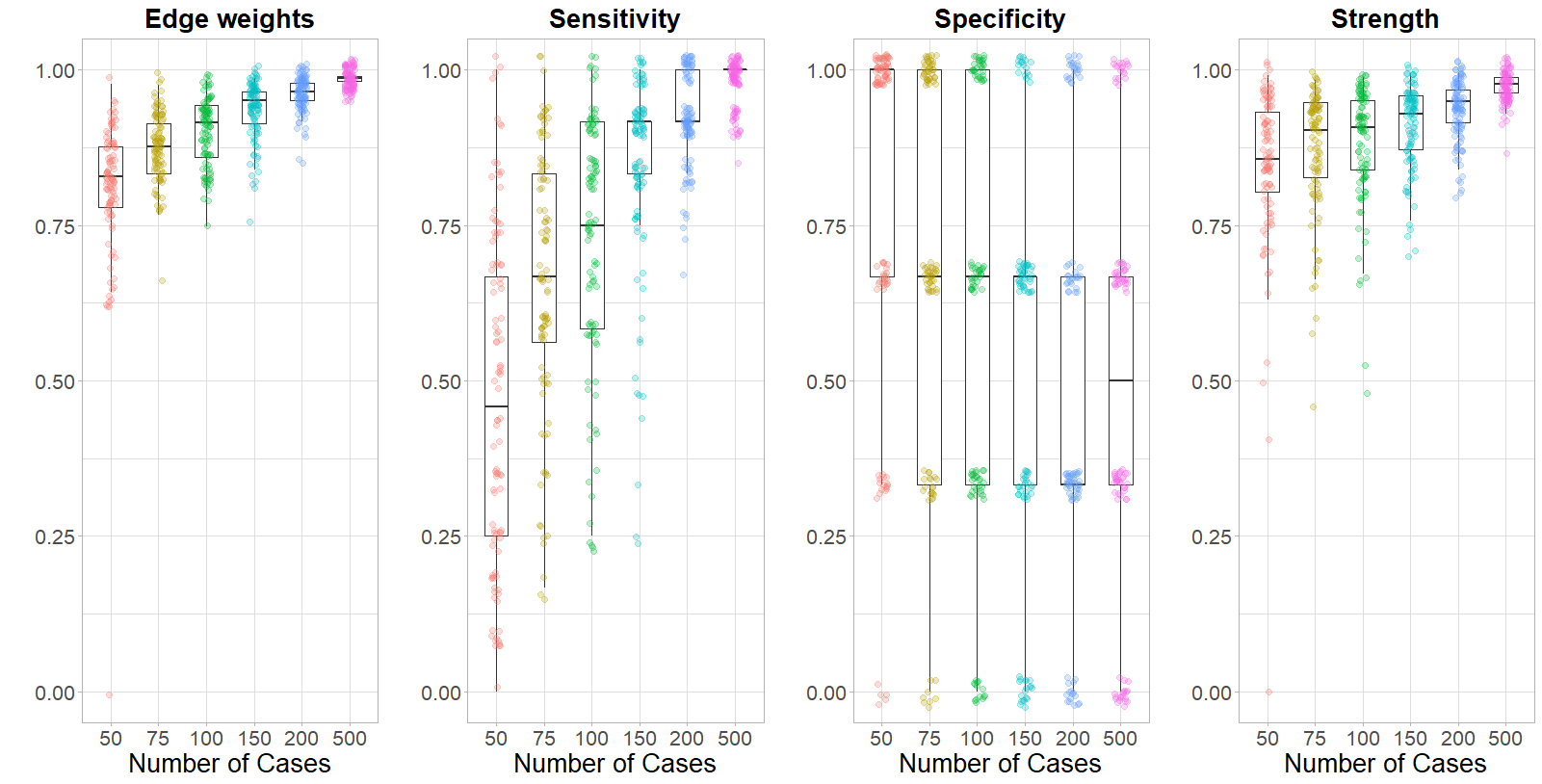


**Supplementary Figure S1.** Estimation of the required sample size. From the data and parameters of the estimated network, the *netSimulator* function allows to set up simulation studies with different sample size and compute the correlations of edge weights, sensitivity, specificity, and correlation of centrality indices between the original and estimated refitted network. The centrality indices strength is presented since it was the only one stable after case-dropping bootstrap. Values of specificity are influenced by the structure of the original network since few edges were set to zero. Overall, 150 number of cases seem to represent an adequate sample size that should be considered for future studies (correlations between original and estimated networks above 0.8).


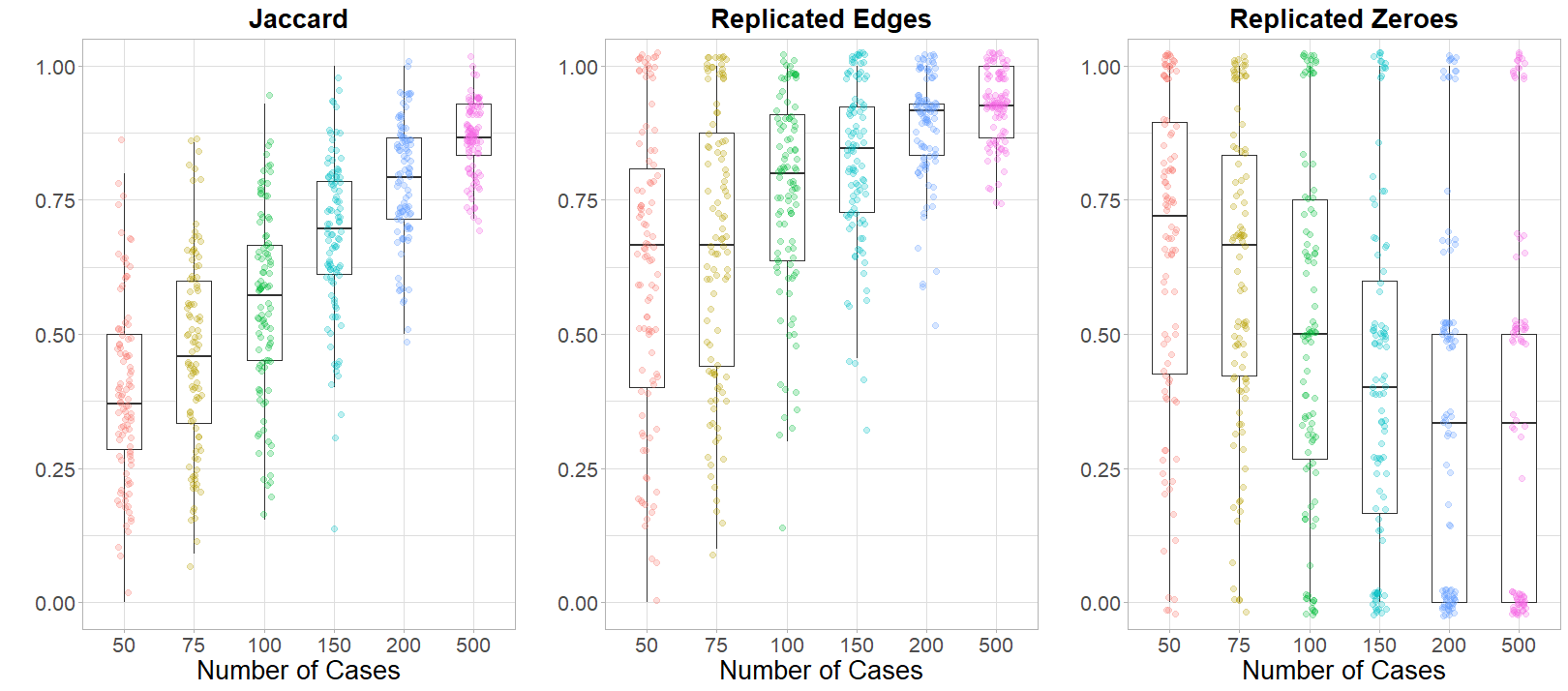


**Supplementary Figure S2.** Replicability of findings using the function *replicationSimulator.* Two datasets from the same model are generated, and the reported values indicate how well the network obtained from the first independent dataset would be replicated in the second one. Again, results are presented for simulated samples with different number of cases. The Jaccard coefficient measures the percentage of overlap of the networks obtained from the two datasets.
